# Extracellular ERp57 promotes fibronectin fibril formation during matrix assembly of articular cartilage

**DOI:** 10.1016/j.isci.2025.114046

**Published:** 2025-11-13

**Authors:** Yvonne Rellmann, Elco Eidhof, Uwe Hansen, Sandra Schulte, Sina Stücker, Thomas Pap, Rita Dreier

**Affiliations:** 1 Institute of Physiological Chemistry and Pathobiochemistry, Waldeyerstraße 15, 48149 Münster, Germany; 2 Institute of Musculoskeletal Medicine, University Hospital Münster, Albert-Schweitzer-Campus 1, Building D3, Münster, Germany

**Keywords:** Biochemistry, Cell biology

## Abstract

Fibronectin 1 (FN1), a general organizer of extracellular matrix (ECM) in various connective tissues, contains disulfide bridges formed by protein disulfide isomerases (PDIs). ERp57 (PDIA3), an ER resident glycoprotein-specific PDI, is also detectable in cartilage extracellular matrix (ECM). Here, we analyzed the extracellular role of ERp57 in FN1 fibrillogenesis in cartilage. ERp57 KO mice exhibited reduced ECM density. Isolated chondrocytes thereof and C28/I2 ERp57 KO chondrocytes formed fewer and shorter FN1 fibrils than WT cells. Significantly, cell membrane-impermeable thiol blockers reduced FN1 assembly in WT cells, while active recombinant ERp57 protein increased it only in the absence of thiol blockers, emphasizing the necessity of ERp57-mediated disulfide bridge formation for FN1 fibrillogenesis. Co-immunofluorescence and proximity ligation assays revealed a direct interaction between ERp57 and FN1. This study highlights a key role for extracellular ERp57 PDI activity in cartilage and further explains phenotypic changes in ERp57 KO animals.

## Highlights

• ERp57 (PDIA3) is detectable in the extracellular matrix surrounding cartilage cells
• ERp57 acts as an extracellular protein disulfide isomerase
• Extracellular disulfide bridge formation by ERp57 is essential for FN1 fibrillogenesis
• The formation of collagen II fibrils is independent of extracellular ERp57 activity

## Graphical abstract

**Figure:**
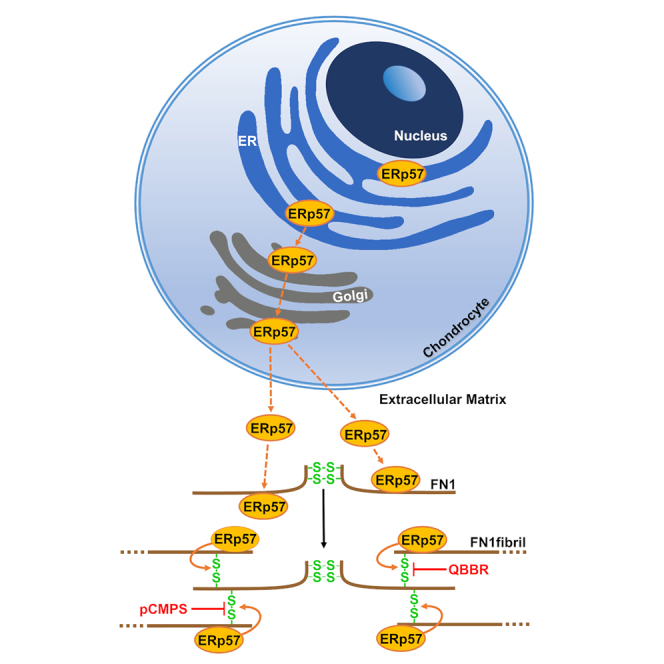

## Introduction

The biomechanical properties of articular cartilage and its function are related to its unique composition and particular structural organization. Chondrocytes, as the only resident cells, make up only a small part of the tissue volume but produce and secrete a large variety of proteins, proteoglycans, and glycoproteins as constituents of the extracellular matrix (ECM) surrounding the cells. The main components of the articular cartilage matrix are collagen II (Col II)-containing cartilage fibrils, which provide tensile strength, and aggrecan, which is assembled into large aggregates by interactions with hyaluronan.^1^ These aggregates contribute anionic charges, which lead to water binding through osmotic effects as a basis for the viscoelastic properties of articular cartilage.^2^^,^^3^

However, other components of the articular cartilage ECM are equally crucial for the structural organization of the tissue and its function. Specific roles of individual components of the cartilage matrix have often been analyzed in studies with knock-out mice^4^^,^^5^ or by means of mutations in genetic cartilage diseases and their correlation with cartilage pathology.^6^ ECM molecules are involved in the assembly of cartilage fibrils and other structural elements, connect different ECM components with each other to form the complex 3-dimensional network, are responsible for the attachment of chondrocytes to the ECM via matrix receptors, act as signaling molecules or their modulators, and influence the metabolic activities of cartilage cells.

Cellular fibronectin 1 (FN1) is a multifunctional ECM glycoprotein expressed by chondrocytes. It actively participates in cell-matrix adhesion and plays a role in the formation and organization of the ECM.^7^^,^^8^ FN1 interacts with many other ECM proteins. It is, for example, reported to be involved in the aggregation of collagen I-containing fibrils^9^ and also displays binding to Col II.^10^ How exactly FN1 is involved in the organization of the cartilage matrix is largely unknown. However, it was shown that FN1 enhances the development of the extracellular filamentous network in newly formed cartilage and increases the accumulation of Col II on the surface of chondrocytes.^11^ Additionally, FN1 activates different signaling pathways in chondrocytes, e.g., signaling via α5β1 and αvβ3 integrins, which enhances PI3K/AKT signaling to promote cartilage matrix formation and chondrogenic differentiation or TGF-β signaling, which can phosphorylate SMAD2/3, leading to enhanced Runx2 expression and release of ECM molecules such as COL2A1 and aggrecan, reviewed in: ^12^.

FN1 monomers of about 250 kDa are formed by repeating domains, the so-called type I, type II, and type III domains. In humans, three exons encode the type III domains, which are termed the extra domains A and B (EDA and EDB) and the variable domain (V). This allows alternative splicing to express different isoforms. In cartilage, three isoforms occur. The isoform (V + C)- accounts for the majority (50–80%) and is exclusively expressed in cartilaginous tissues.^13^ In addition, 20–30% of the isoform EDB(+) and less than 4% of the isoform EDA(+) exist.^14^ Although most FNs have been shown to be secreted as disulfide-linked dimers of about 440 kDa, the (V + C)-FN1 isoform was mainly secreted as a monomer in a mammalian expression system and has been reported to be less effective than other FN1 isoforms at cell adhesion and binding to integrin α5β1 but more active at binding to chondroitin sulfate E. However, to form FN1 fibrils, FN1 dimerization is essential.^15^ Whether this dimerization of FN1 can also occur extracellularly is not yet known. Within FN1 dimers, two disulfide bridges connect the monomers near the C-termini (two S-S bonds), and others fold and stabilize type I and type II domains. The type III domains lack stabilizing disulfide bridges and are therefore able to allow conformational changes due to mechanical forces.^16^

Mutations in the human FN1 gene are associated with the skeletal disease spondylometaphyseal dysplasia with “corner fractures.”^17^ Such mutations often affect cysteine residues.^18^ The exchange of cysteine residues for other amino acids most likely leads to impaired protein folding by protein disulfide isomerases (PDIs) in the endoplasmic reticulum (ER). In cartilage, this can lead to ER stress and reduced secretion of FN1 into the ECM.^16^^,^^19^ In other tissues, e.g., in the kidney, mutated FN1 was detected extracellularly, but due to its defective structure and abnormal interactions with heparin, it was apparently unable to form normal FN1 fibrils.^20^

The mechanism of FN1 fibrillogenesis seemed to be clear for a long time. It was assumed that FN1-secreting cells build at their surfaces long ropes of elongated FN1 dimers in which N-terminal domains overlap alternately with regions containing C-termini.^21^ During the assembly, fibronectin undergoes conformational changes that expose fibronectin-binding sites and promote intermolecular interactions needed for fibril formation.^22^ Recently, however, a new paradigm of FN1 fibrillogenesis has been established using live imaging and single-molecule localization microscopy. These analyses revealed that FN1 fibrils tend to consist of spherical nanodomains containing six to eleven FN1 dimers organized in linear arrays.^23^ Both models assume that FN1 fibrillogenesis depends on FN1 dimers, that FN1 fibrillogenesis occurs at the cell surface of producing cells, e.g., by binding to integrins,^24^ and that the cytoskeleton exerts traction forces on FN1 dimers that expose epitopes for FN1 interactions to form the fibrils.^25^ The interacting epitopes are most likely only present and functional if FN1 dimers exhibit perfect protein folding with intact disulfide bridges at the correct positions. Therefore, the disulfide bridge-forming protein disulfide isomerases (PDIs), which are responsible for the post-translational modification and folding to enable subsequent FN1 secretion, are crucial for the extracellular presence of fibrillogenesis-competent FN1.

The family of PDIs includes 21 members^26^ that catalyze the formation, cleavage, and isomerization of protein disulfide bonds between cysteine residues within protein chains during protein folding. Among these, there are five membrane-bound PDIs grouped in the thioredoxin-related transmembrane (TMX) protein family, known as TMX1-5.^27^ This study focuses on endoplasmic reticulum protein 57 (ERp57), also known as PDIA3, as it exhibits substrate specificity for glycoproteins due to its interaction with the lectins calnexin and calreticulin.^28^^,^^29^^,^^30^^,^^31^ A large proportion of cartilage proteins, including FN1 and Col II, are glycoproteins and therefore possible substrates of ERp57.

Initially identified as proteins of the endoplasmic reticulum, ERp57 and other PDIs have also been reported to reside on the cell surface and in the ECM.^26^^,^^32^^,^^33^ While the chaperone function of ERp57 in the ER has been clearly demonstrated in cartilage-specific ERp57 knock-out (KO) mice by misfolding of proteins with protein aggregation in the ER and resulting ER stress that impairs the function of the growth plate and articular cartilage,^34^^,^^35^ a possible extracellular function of ERp57 in cartilage has not yet been analyzed.

This study elucidates the extracellular function of ERp57 in FN1 fibrillogenesis and its impact on the overall structure of the ECM in articular cartilage. Because it is known that a KO of ERp57 in mice leads to accelerated osteoarthritis (OA) development in knee joints,^35^ these data may also provide valuable information for the understanding of OA formation.

## Results

### ERp57 knockout mice mice display severe structural extracellular matrix defects in the knee joint cartilage

**Figure 1:**
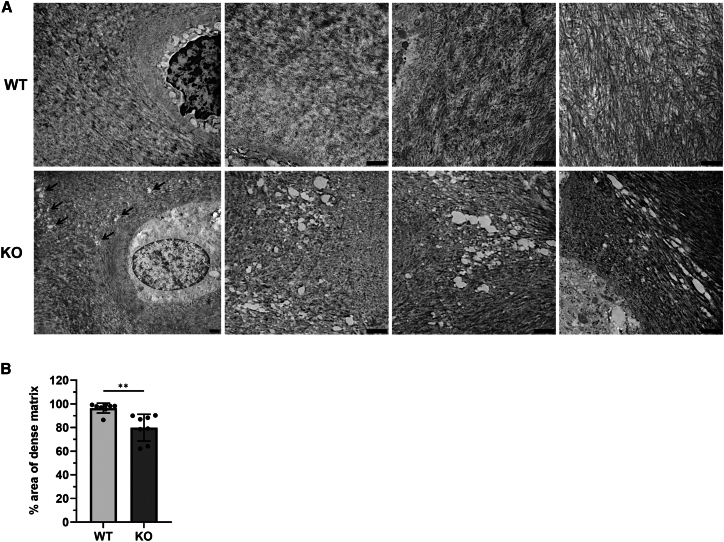
ERp57 KO mice display severe structural ECM defects in knee joint cartilage High-magnification transmission electron microscopic (TEM) analysis of articular cartilage isolated from 18-week-old WT and ERp57 KO mouse knees. KO samples exhibit a significantly lower ECM density around chondrocytes with holes in the territorial/interterritorial matrix (marked with arrows) (A). In microphotographs of WT samples, an average of 96% of the total area was covered with dense matrix, compared to 79% in the KO (B). Statistical evaluation was performed with Student’s *t* test. Data are mean ± SD. ∗∗ represents a *p*-value of <0.01. N (number of animals per genotype) ≥ 4; n (number of analyzed images per genotype) = 8; scale bars = 1 μm.

To investigate whether the knockout of the PDI ERp57 in chondrocytes has an effect on the ECM structure of knee joint cartilage, tissue samples from WT and KO animals (Table S1) were analyzed and compared using transmission electron microscopy (TEM). In the examined cartilage samples from adult male and female WT mice, the articular cartilage cells are embedded in a dense and well-structured cartilage ECM (Figure 1A, top panel). In contrast, knee cartilage samples from male and female cartilage-specific ERp57 KO animals revealed a significantly lower ECM density. In ERp57 KO cartilage, some areas between the chondrocytes contained no fibrillar meshwork, so that holes were detectable in the examined ultrathin sections (Figure 1A, bottom panel, holes marked with arrows). The holes were found in the interterritorial matrix, where normally a dense ECM with thick Col II bundles is responsible for the mechanical properties, such as the tensile strength of the articular cartilage.^36^ Whether the pericellular matrix is also affected is not apparent in TEM analysis. Additional immunofluorescence analyses of articular WT and ERp57 KO cartilage did not provide further information to distinguish between the effects of ERp57 on FN1 fibrillogenesis in pericellular and interterritorial zones (Figure S1). The image analysis quantifying the area of dense matrix in WT and ERp57 KO knee cartilage (Figure 1B) revealed a 17% reduction in ERp57 KO animals compared to WT mice. In other words, 17% of the area in the ERp57 KO cartilage images shows holes in the ECM.

### Primary ERp57 knockout mice chondrocytes produce less fibrillar matrix in micromass cultures

**Figure 2:**
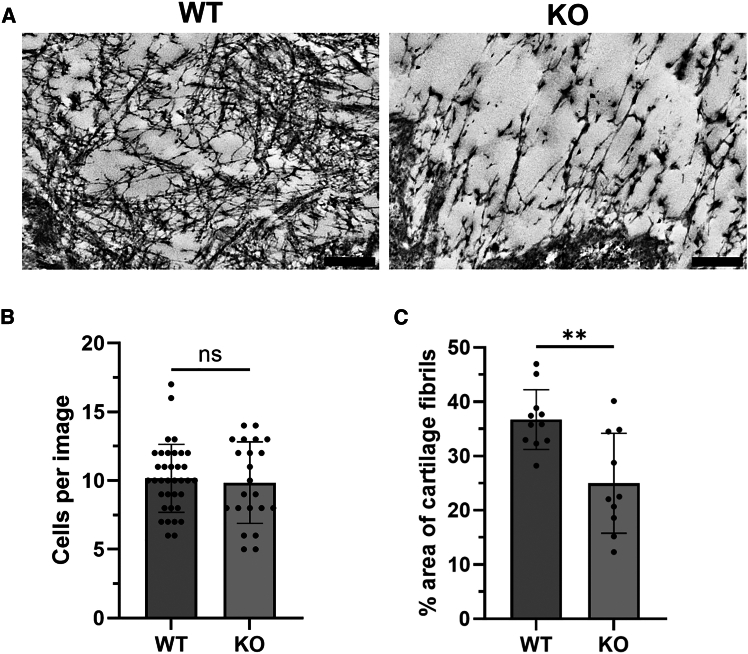
Primary ERp57 KO chondrocytes produce less fibrillar matrix than WT cells Transmission electron microscopic (TEM) analysis of micromass cultures of primary WT and ERp57 KO chondrocytes isolated from knee joints of newborn mice revealed fewer and shorter cartilage fibrils in KO samples compared to WT controls (A), although the cell number is comparable (B). In microphotographs of WT samples, an average of 37% of the total area was covered with fibrils, compared to 25% in the KO (C). Statistical evaluation was performed with Student’s *t* test. Data are mean ± SD. ∗∗ represents a *p*-value of <0.01. ns indicates non-significant *p*-values. N (number of animals per genotype) = 4; n (number of micromasses per genotype) ≥ 10; scale bars = 500 nm.

Next, micromass cultures of primary WT and ERp57 KO chondrocytes isolated from these mouse strains (Table S1) were examined to compare the formation of new cartilage fibrils *in vitro*. At the endpoint after 7 days of culture, the micromasses showed equal cell numbers of WT and KO cells (Figure 2B), equal FN1 protein levels (Figure S2), but very different ECM structures in the TEM analysis (Figure 2A). The WT cells (Figure 2A, left panel) produced a matrix with many long cartilage fibrils, while the ERp57 KO cells (Figure 2A, right panel) showed many fewer and shorter fibrillar structures. Image analysis was used to compare the mean percentage of extracellular area filled with cartilage fibrils within WT and ERp57 KO samples after 7 days of culture (Figure 2C). In micromasses of WT cells, 37% of the total area was covered with cartilage fibrils, whereas in ERp57 KO micromasses only 25% of the total area contained fibrillar structures. These results suggest that ERp57 is specifically involved in fibril formation in the cartilage ECM.

### Cultured C28/I2 ERp57 knockout mice cells exhibit a reduced extracellular network of fibronectin 1

**Figure 3:**
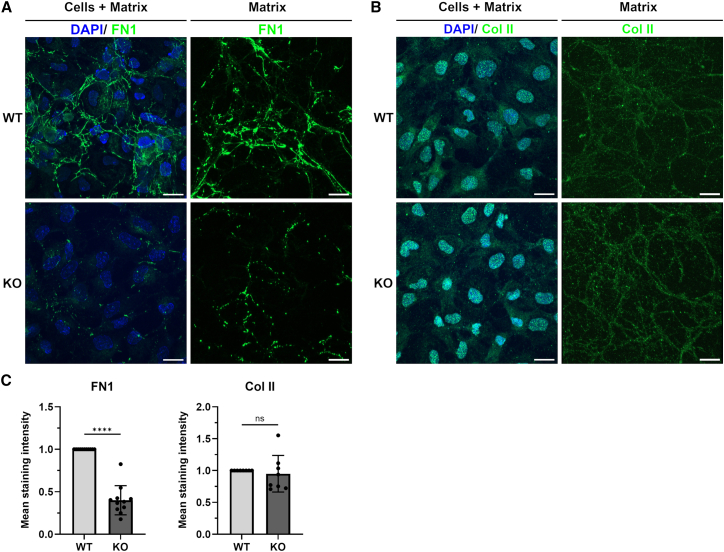
Cultured C28/I2 ERp57 KO cells exhibit a reduced extracellular network of fibronectin 1 but unchanged collagen II fibrils Immunofluorescence analyses of the extracellular matrix (ECM) produced by C28/I2 WT and C28/I2 ERp57 KO chondrocytes, examined after fixation (Cells + Matrix) or after decellularization and fixation (Matrix) to visualize the ECM fibrils without cell-derived signals. The figure shows the projections of z-stacks. Punctate Col II signals in non-decellularized samples (Cells + Matrix) reveal Col II-containing vesicles near/above the nuclei of chondrocytes. Fibronectin (FN1) and collagen II (Col II) fibrils were detected in WT samples, including cells and matrix, and also in decellularized samples containing only matrix. The FN1 network was significantly reduced in KO samples (A). In contrast, the Col II network was comparably well developed in ERp57 KO and WT cells (B). Quantitative analysis of the decellularized samples revealed a reduction in the mean staining intensity of the FN1 matrix by more than 60% in the KO samples compared to WT controls and no statistically significant difference in Col II staining in samples of both genotypes. (C) Statistical evaluation was performed with the Student’s *t* test. Data are mean ± SD. ∗∗∗∗ represents a *p*-value of <0.0001, ns indicates non-significant *p*-values. *N* ≥ 8 (number of experiments), *n* ≥ 30 (technical replicates). Scale bars = 20 μm.

To analyze how the formation of the extracellular fibrillar network in cartilage depends on ERp57, C28/I2 cells, a human chondrocyte cell line commonly used as a model for the biology and physiology of normal and pathological chondrocytes,^37^ were used as WT and as CRISPR/Cas9-generated ERp57 knockout versions^38^ in monolayer cultures. Immunofluorescence analyses were performed to detect FN1 and Col II in samples containing cells with surrounding ECM (Cells + Matrix) and in decellularized samples containing ECM only (Matrix). Regions with similar cell densities were analyzed. The comparable number of stained cell nuclei (DAPI staining) (Figures 3A and 3B, left panels and S3) indicates an equally high vitality and proliferation in both genotypes. WT samples showed FN1 fibrils forming a dense network around the C28/I2 cells, which is visible on the slides with cells, but becomes even more obvious in the decellularized samples (Figure 3A, top). ERp57 KO cells, on the other hand, displayed a barely visible FN1 matrix with only very thin and short fibrillar structures (Figure 3A, bottom). The statistical evaluation of the results revealed a reduction in the mean staining intensity of FN1 positive structures by 60%. To support the extracellular role of ERp57 in this context, various control experiments were performed. A reduced secretion of FN1 by C28/I2 ERp57 KO cells compared to WT cells was excluded by immunoblotting of cell lysates and culture media of WT and ERp57 KO cells. The data revealed a comparable amount of secreted FN1 in samples of both genotypes (Figure S4) with similar levels of monomeric or dimeric FN1 (Figure S5), suggesting that the dimerization of FN1 in ERp57 KO cells is mediated by a different PDI. We have additionally observed that PDIA1 (PDI) is present in ERp57 KO C28/I2 cells but cannot rescue the FN1 fibrillogenesis (Figure S6). To exclude the possibility that ER stress has a significant impact on FN1 fibrillogenesis, the chemical chaperone 4-Phenylbutyric acid (4-PBA) and the ER stress inductor Thapsigargin were added to WT and ERp57 KO cultures. Both agents had no effect on FN1 fibrillogenesis (Figure S7). Similar immunofluorescence analyses were used to evaluate the density of Col II structures in newly formed ECM. Both cell types (WT and ERp57 KO cells) formed a compact network around the C28/I2 cells. Statistical analysis of these data (Figures 3B and 3C) revealed comparable staining intensities in WT and KO samples, indicating that the Col II network forms independently of ERp57. These data additionally displayed that WT and KO cells are comparably vital and productive. Overall, our analyses indicate an ERp57-dependent formation of the fibrillar FN1 network but not of Col II structures in the analyzed cartilage cell cultures.

### The formation of disulfide bridges is essential for fibronectin 1 but not for collagen II fibrillogenesis

**Figure 4:**
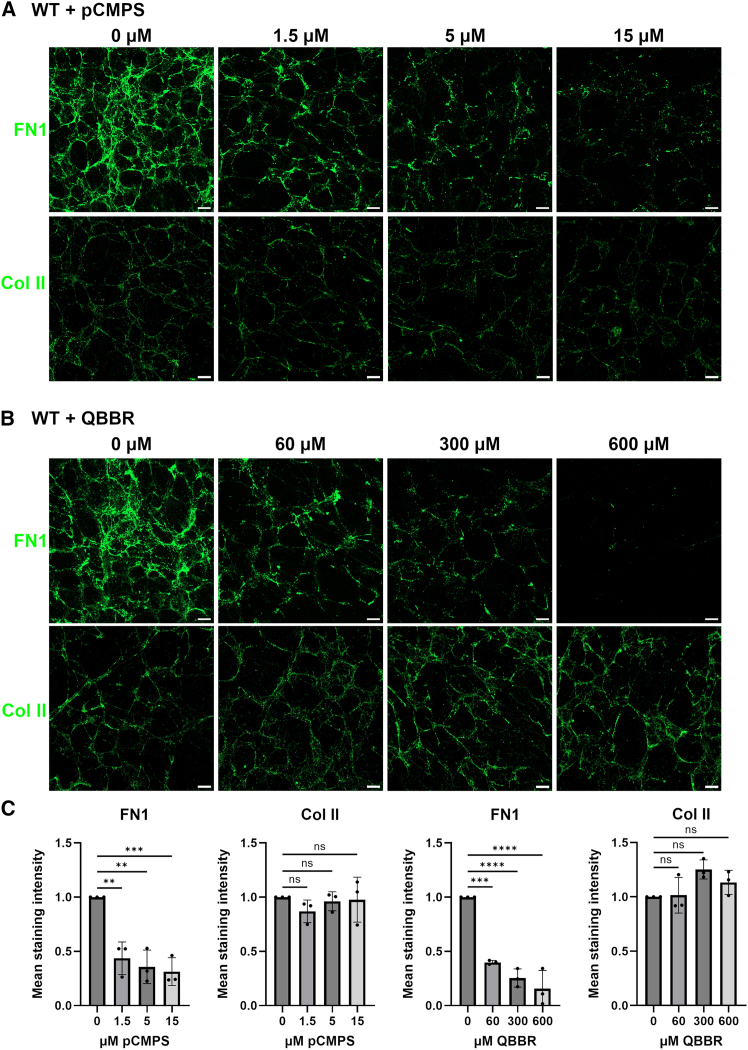
The formation of disulfide bridges is essential for fibronectin 1 but not for collagen II fibrillogenesis Immunofluorescence analyses of FN1 and Col II fibrils produced by C28/I2 WT chondrocytes cultured for 72 h in the presence of non-cell-permeable thiol-blocking agents *p*-Chloromercuriphenylsulfonate (pCMPS) (1.5, 5, and 15 μM) (A) and Monobromo(trimethylammonio)bimanbromide (QBBR) (60, 300, and 600 μM) (B) to inhibit extracellular protein disulfide isomerase activity. The samples were examined after decellularization and fixation. The quantitative analysis of the mean staining intensities of the fluorescence signals revealed a reduction of (long) FN1 fibrils with increasing concentrations of both thiol-blocking agents, while the Col II network was not impaired (C). Statistical evaluation was performed with ordinary one-way ANOVA with Tukey’s post-hoc-test. Data are mean ± SD. ∗∗∗∗ represents a *p*-value of *p* < 0.0001 ∗∗∗ represents a *p*-value of *p* < 0.001, ∗∗ represents a *p*-value of *p* < 0.01, ns indicates non-significant *p*-values. *N* = 3 (number of experiments), *n* ≥ 8 (technical replicates). Scale bars = 20 μm.

Since ERp57 can form (oxidize), break (reduce), and rearrange (isomerize) disulfide bonds, the formation of extracellular disulfide bridges during the fibrillogenesis of FN1 was investigated. Two thiol blocking agents, Monobromo(trimethylammonio)bimanbromide (QBBR) and *p*-Chloromercuriphenylsulfonate (pCMPS), which do not readily penetrate cell membranes,^39^^,^^40^ were used to elucidate the extracellular effects of ERp57 in C28/I2 WT cell cultures. pCMPS dose-dependently inhibits the FN1 fibril formation in monolayer cultures (Figure 4, top). A concentration of 1.5 μM pCMPS was sufficient to achieve a significant reduction in the FN1 network around the cells. The mean staining intensity in decellularized samples was reduced by 56%. 15 μM pCMPS reduced the staining intensity of FN1 fibrils by 69% (Figure 4C, left panel). Only a few, very short FN1 fibrils were still detectable (Figure 4, top right). A comparable effect was obtained in cells cultured with QBBR (Figure 4B, top, with statistical evaluation shown in Figure 4C). 60 μM and even stronger 300 μM, clearly reduced the newly formed FN1 network. In the presence of 600 μM QBBR, hardly any FN1 fibrils were detectable, and the mean staining intensity was significantly reduced by 84%. To rule out changes in FN1 secretion upon treatment with the used thiol blocking agents, immunoblotting of cultured cells and conditioned cell culture media was used. Neither pCMPS nor QBBR reduced the secretion of FN1 by the cells (Figures S8A and S8B). These results clearly indicate that extracellular disulfide bridge formation by PDIs, e.g., by ERp57, is essential during the formation of the FN1 network in the ECM around cartilage cells. In contrast, Col II network formation was not impaired in WT C28/I2 cells in the presence of the thiol blocking agents pCMPS (Figure 4A, bottom) and QBBR (Figure 4B, bottom), with statistical evaluation shown in Figure 4C. This shows that the used concentrations of the thiol blockers pCMPS and QBBR have no toxic effects on cartilage cells and thus do not bias the interpretation of the results. This is in line with the comparable number of DAPI-stained cells in all simultaneously cultured but not decellularized samples (Figure S9).

### Extracellular ERp57 interacts directly with fibronectin 1 fibrils

**Figure 5:**
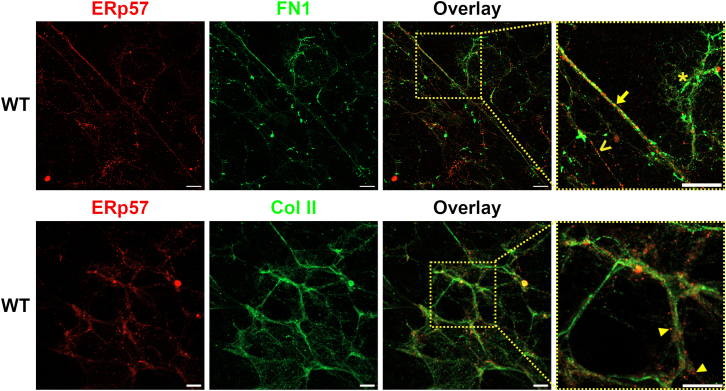
Extracellular ERp57 colocalizes with fibronectin 1 fibrils Co-Immunofluorescence analysis of FN1/ERp57 (A, top panel) and Col II/ERp57 (B, bottom panel) on decellularized matrices. In C28/I2 WT samples, ERp57 was detected on FN1 fibrils in different quantities (← ERp57 high, FN1 high, < ERp57 high, FN1 low, ∗ ERp57 low, FN1 high). The Col II network showed no direct colocalization with ERp57, however ERp57 was detectable in close vicinity to Col II structures (◄). *N* = 3. Scale bars = 20 μm.

**Figure 6:**
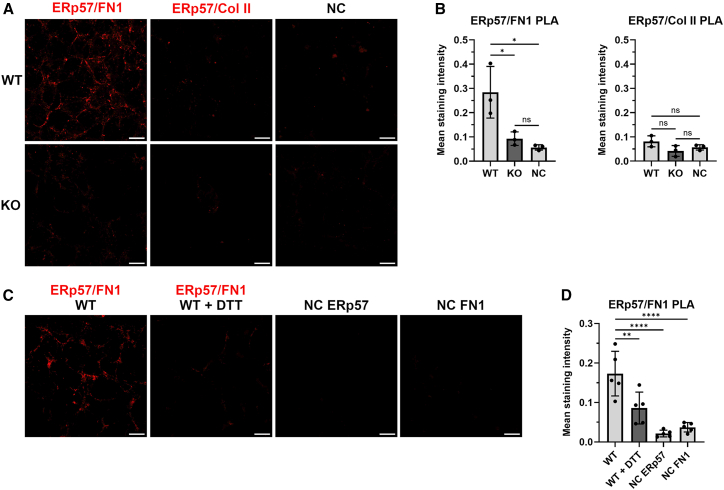
Extracellular ERp57 interacts directly with fibronectin 1 fibrils Proximity ligation assays (PLA) showed FN1/ERp57 interactions, visible as red dots on fibrillar structures of the extracellular matrix (ECM) (A). The corresponding statistical analysis (B) revealed a mean staining intensity of 0.284 ± 0.1065, which differed significantly from the mean staining intensities in the matrix of ERp57 KO cells and in the negative control (WT matrix without primary antibodies). In contrast, no interactions between Col II and ERp57 were detectable using PLA. The mean staining intensity in the WT-produced ECM did not exceed the background staining of the fibrils produced by ERp57 KO cells or the negative control (WT matrix without both primary antibodies). Short-term incubation with the reducing agent dithiothreitol (DTT) reduced PLA signals (C and D) significantly. Omission of ERp57 or FN1 antibodies reduced PLA signals to background levels (D). Statistical evaluation was performed with one-way ANOVA with Tukey’s post-hoc-test. Data are mean ± SD. ∗ represents a *p*-value of <0.05. *N* = 3 (number of experiments), *n* = 12 (technical replicates). Scale bars = 20 μm.

In case extracellular ERp57 forms disulfide bridges to establish the FN1 network, a direct interaction between ERp57 and FN1 is required. To detect such contacts, WT monolayers were decellularized, and the matrices were analyzed by co-immunofluorescence staining with antibodies against FN1 and ERp57 and examined by confocal microscopy. As a control, co-immunofluorescence staining of ERp57 and Col II was performed (Figure 5). In decellularized matrices, FN1 fibrils were visible along their entire length and showed colocalization of FN1 and ERp57, although the intensity of ERp57 signals differed for various fibrils. Some FN1 fibrils, such as the long straight fibril in the middle of the image (Figure 5 top panel, and enlarged overlay image section on the top far right, highlighted with →), showed a very high concentration of ERp57 colocalized with FN1, with comparable signal intensity for FN1 and ERp57. Few FN1-positive fibrils showed only low ERp57 signal intensity (∗), while some ERp57-positive fibrils were almost free of FN1 (>). The lack of ERp57 staining on some FN1 fibrils indicates that the ERp57 antibody does not cross-react with FN1 protein. The specificity of the used ERp57 antibody was additionally demonstrated by immunoblot analyses (Figure S10). In contrast, Col II-containing fibrils showed no direct colocalization of Col II and ERp57. In all images, ERp57 occurred in the close vicinity of Col II-stained structures (marked with arrowhead), but not as part of these fibrils. In addition, a proximity ligation assay (PLA)^41^ clearly demonstrated the direct protein interaction of ERp57 and FN1, but not of ERp57 and Col II on fibrillar structures produced by C28/I2 WT cells (Figures 6A and 6B). In the matrix of C28/I2 ERp57 KO cells, PLA signals were, as expected, low and comparable to the background signals of the negative controls (NC, WT cells without both primary antibodies). Comparable densities of the matrix used in the PLA assays were confirmed by DAPI staining (see Figure S11). To confirm that the effect of recombinant ERp57 is attributed to its redox activity, an additional PLA after short-time incubation (15 min) with dithiothreitol (DTT) was performed. The presence of this reducing agent significantly decreased the signal intensity (Figures 6C and 6D), suggesting that ERp57 only binds to FN1 structures in its oxidative form. As expected, the negative controls omitting ERp57 or FN1 antibodies showed only negligible background staining (Figures 6C and 6D).

### Active recombinant ERp57 protein added to the culture medium increases fibronectin 1 fibrillogenesis around ERp57 knockout mice cells *in vitro*

**Figure 7:**
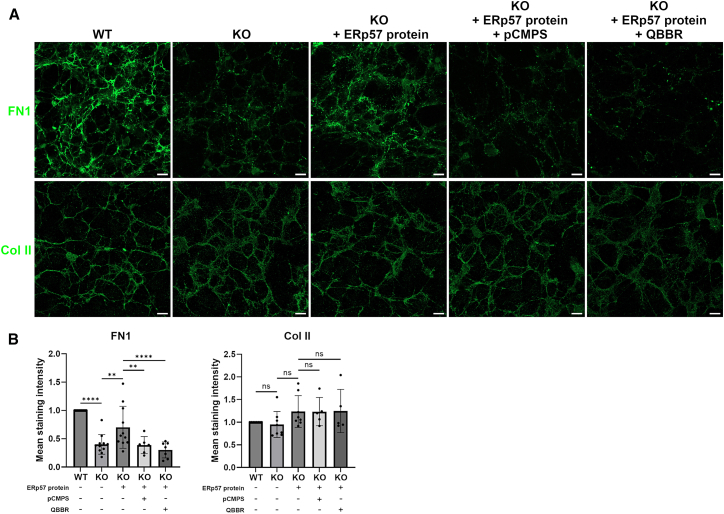
Active recombinant ERp57 protein added to the culture medium increases the fibronectin 1 fibrillogenesis around ERp57 KO cells *in vitro* Immunofluorecence analysis of FN1 and Col II on decellularized matrices of C28/I2 WT and C28/I2 ERp57 KO cells. Some of the KO cells were cultured for the entire culture period of 72 h in the presence of 0.1 μM active recombinant ERp57 protein or in the presence of 0.1 μM active recombinant ERp57 protein with the addition of 5 μM *p*-Chloromercuriphenylsulfonate (pCMPS) or 300 μM Monobromo (trimethylammonio) bimanbromide (QBBR) (A). KO cells showed in the quantitative analysis a strongly reduced staining intensity of FN1 and an unchanged staining intensity of Col II (B). The addition of active recombinant ERp57 protein to the cell culture medium of KO cells led to an increase in the mean staining intensity of FN1 (partial rescue), which was reduced again by the simultaneous addition of pCMPS and QBBR. The staining intensity of Col II was not significantly affected by the addition of active recombinant ERp57 protein in the presence or absence of pCMPS or QBBR. Statistical evaluation was performed with two-way ANOVA with Tukey’s post-hoc-test. Data are mean ± SD. ∗∗∗∗ represents a *p*-value of *p* < 0.0001, ∗∗ represents a *p*-value of *p* < 0.01, ns indicates non-significant *p*-values. *N* ≥ 5 (number of experiments), *n* ≥ 16 (technical replicates). Scale bars = 20 μm.

In the key experiment to demonstrate the extracellular role of ERp57 in FN1 fibrillogenesis, active recombinant ERp57 protein was added to ERp57 KO cell cultures. However, the concentration of secreted ERp57 in cartilage is not yet known. We assume that under the applied cultivation procedure, less than 10% of the total ERp57 protein is released from a chondrocyte into the ECM (see Figure S12). Further details on this issue will be obtained in current and future experiments, which will also analyze the possible stimuli or conditions for ERp57 secretion, such as ER stress, senescence, or inflammation. We added commercially active recombinant ERp57 protein for which the manufacturer has tested activity and purity by measuring insulin aggregation in the presence of DTT and SDS-PAGE analysis. The potential rescue of FN1 fibrillogenesis was subsequently analyzed by immunofluorescence staining with quantitative image analysis of fibrillar structures present after 72 h of culture. In addition, the previously used extracellularly acting thiol blockers (pCMPS and QBBR) were used in some samples to block the PDI activity of the added recombinant ERp57 protein. Fibrillogenesis of FN1 was significantly increased after the addition of 0.1 μM ERp57 to the cell culture medium (Figure 7A). When the percentage of FN1-positive areas in the microphotographs of the stained WT samples was set to 100%, this value was reduced to 40% in the KO cells and increased to 70% in the KO cells cultured in the presence of recombinant ERp57 protein (Figure 7B). The image analysis thus indicated a partial rescue of FN1 fibrillogenesis by extracellularly supplied ERp57. And this change is indeed due to the PDI activity of ERp57, as the thiol-blocking agents pCMPS and QBBR reduced these values to 30 and 24%, respectively. During the culture period of 72 h added ERp57 is not taken up by the cells through endocytosis, as demonstrated by additional experiments shown in Figure S13, emphasizing the extracellular effect of ERp57. As a control, the formation of the Col II network was also analyzed. The addition of ERp57 to the medium of cultured KO cells did not have any significant effect on Col II fibrillogenesis. In Col II staining, the values were 100% in WT cells, 95% in KO cells, 124% in KO cells cultured in the presence of ERp57, 115% in KO cells, cultured in the presence of ERp57 and pCMPS, and 122% in KO cells, cultured in the presence of ERp57 and QBBR. This indicates that Col II fibrillogensis is not dependent on the formation of extracellular disulfide bridges by ERp57.

## Discussion

In the present study, the PDI ERp57 was detected in significant amounts outside of cartilage cells within the ECM. Normally, this PDI is localized in the ER, where it interacts with the lectins calreticulin and calnexin to fold newly synthesized glycoproteins.^42^^,^^43^ All PDI family members contain a highly acidic tetrapeptide ER retention signal. The KDEL (Lys-Asp-Glu-Leu) motif is recognized by KDEL receptors of the Golgi apparatus in order to transport the binding proteins back into the ER via COP I vesicles. However, variations in this motif (e.g., KTEL, EDEL, and QSEL) suggest different affinities to KDEL receptors and thus different PDIs may escape the ER to be secreted into the extracellular space.^44^ ERp57, also called PDIA3, contains a QEDL sequence^45^ as a further variant of the KDEL motif and was detected in the medium of transformed NIH3T3cells by Hirano et al.,^32^ in cell culture supernatants of renal cells by Dihazi et al.,^33^ and in the medium of cultured cartilage cells by us.^46^ Interestingly, it was recently observed that a subgroup of KDEL receptors is able to transport PDI family members (e.g., PDIA1, PDIA3, and PDIA6) to the plasma membrane.^47^ From there, ERp57 could be released into the extracellular space. The possibility of KDEL receptor-mediated secretion of ERp57 from the ER via the Golgi apparatus into the ECM of cartilage or alternative secretory mechanisms should be investigated in detail in the future.

However, in our cartilage cell culture system, ERp57 is not only present outside of chondrocytes but is also enzymatically active there and directly involved in fibril formation in the cartilage ECM. We observed that the loss of extracellular ERp57 led to a significant decrease in FN1 fibrils. Previously, a reduced fibronectin content had been detected in fibroblasts after functional disruption or silencing of ERp57, but this phenomenon was attributed to the impaired intracellular chaperone function of ERp57 within the ER.^48^ In contrast, we have shown here that it is the extracellular ERp57 that is essential for FN1 fibrillogenesis. Whether extracellular ERp57 activity depends on calnexin or calreticulin was not included in the study and should be investigated in the future. In the context of tumor development, Ros et al. found that ERp57-calnexin complexes reduce extracellular disulfide bonds and are essential for ECM degradation.^49^ ERp57 binds to FN1 fibrils in the extracellular space and exerts its PDI activity there. FN1 fibrillogenesis is significantly inhibited in the presence of plasma membrane-impermeable thiol blockers and at least partially rescued in KO cells by the addition of active recombinant ERp57 protein. Whether other members of the PDI family, e.g., the membrane bound TMX proteins,^27^ also exhibit extracellular enzyme activity remains to be determined.

In cartilage, fibrillar FN1 is present throughout chondrogenic differentiation, but it is also retained in mature cartilage tissue, such as the growth plate.^50^ Initially, FN1 is rapidly upregulated during the condensation of mesenchymal cells.^51^ In the early stages of chondrogenesis, the first FN1 fibrils are formed, and Col I is assembled. This pre-cartilaginous ECM is subsequently replaced by meanwhile differentiated chondrocytes producing Col II and aggrecan. In mature cartilage, FN1 is involved in cartilage repair, where it most likely activates progenitor cells.^52^ It also promotes chondrocyte differentiation and collagen production via the TGF-β/PI3K/Akt pathway, as shown in mice with femoral fractures.^53^

In previous studies, we analyzed ERp57 effects in different cartilage tissues and observed that it is critically involved in growth plate function^34^ and articular cartilage homeostasis.^35^ Interestingly, cartilage-specific ERp57 KO mice showed reduced long bone growth,^34^ a phenotype that was similarly observed in double KO mice for cellular and plasma FN isoforms (FNdKO).^54^ Furthermore, a reduction in trabecular bone parameters and an elongation of hypertrophic growth plate zones were observed in both ERp57 KO and FNdKO samples. Overall, the skeletal phenotype observed in the ERp57 KO model shows strong similarities to that of the FN isoform KO in the skeletal system, supporting our data on the involvement of ERp57 in FN1 fibrillogenesis. We also examined the knee joints of cartilage-specific ERp57 KO mice, which revealed reduced compressive stiffness of articular cartilage and accelerated OA pathogenesis with early osteophyte formation after loss of ERp57 in chondrocytes.^35^^,^^55^ At that time, however, we focused on the intracellular chaperone function of ERp57. It is now becoming increasingly clear that the loss of extracellular ERp57 and its effects on FN1 fibrillogenesis are also likely to have a major impact on the OA phenotype of ERp57 KO mice.

With regard to FN1, cartilage is unique because the protein occurs in a special splice variant. A large proportion (50–80%) of FN1 in human cartilage is composed of (V + C)^-^ FN, and interestingly, this FN isoform exists as homodimers but also in an unusual monomeric configuration called single-chain fibronectin.^14^^,^^56^^,^^57^ To our knowledge, it has not yet been investigated whether such monomers are incorporated into fibrillar structures at all and, if so, which mechanisms are involved. Future studies will elucidate whether extracellular PDIs such as ERp57 are involved.

FN1 is regarded as a master organizer of the ECM because the deposition and assembly of fibrillar collagens and many other ECM proteins in various tissues depend on a pre-assembled FN1 network.^11^^,^^58^ Whether and which ECM molecules are possibly affected in ERp57 KO mice must be analyzed in detail in future studies. However, fibronectin fibrils are not only essential for the structural integrity of cartilage tissues but also form ligands for matrix receptors of the integrin family. Normal adult articular chondrocytes express α1β1, α3β1, α5β1, α10β1, αVβ1, αVβ3, and αVβ5 integrins,^59^ with α5β1, αvβ3, and αvβ5 integrins binding fibronectin, thereby mediating the adhesion of chondrocytes to the ECM and initiating intracellular signaling pathways.^60^ Dihazi et al.^33^ and Hellewell et al.^48^ demonstrated reduced cell spreading and less focal adhesion- and F-actin assembly when ERp57 was inhibited or knocked out. These observations could be due to a lack of FN1 fibrils bound to integrins or to a lack of activation of the integrins themselves, as integrin β1 has also been shown as an ERp57 substrate in a substrate trapping approach.^30^ In some instances, different PDIs are involved in integrin activation. For example, in platelets, integrins interact with PDI, ERp57, and ERp72, and each enzyme plays an individual role in platelet aggregation to promote thrombosis.^61^

Our results are of great interest for orthopedic research, as alterations in FN1 or in the FN1-triggered activation of integrins, both of which we assume to depend on extracellular ERp57 activity, are associated with skeletal diseases such as OA or spondylometaphyseal dysplasia (SMD) with corner fractures.^16^

During OA, significant changes occur in the FN1 transcriptome that impair the proper structural function of the protein as well as the activation of the integrin. For example, a truncated FN1-208 transcript, which lacks the RGD integrin binding site, is significantly upregulated. The increased FN1-208:FN1 ratio leads to the decreased deposition of soluble glycosaminoglycans (sGAGs) as well as decreased aggrecan (ACAN) and col II (COL2A1) and increased aggrecanase (ADAMTS-5) and integrin (ITGB1 and ITGB5) gene expression.^62^ All these changes are typical features of OA cartilage. However, whether an interaction of FN1-208 with ERp57 is still possible and what effects this might achieve remains an open question.

Mutations in FN1, which in most cases affect cysteine residues, are the cause of SMD with corner fractures.^16^ According to our results, such mutations would prevent the formation of disulfide bridges by PDIs, especially ERp57. This would result in incorrect protein folding in the ER with protein aggregation and ER stress (16), but also in a reduced incorporation of FN1 molecules into extracellular FN fibrils. Short and thin FN1 fibril aggregates would be formed, as observed in cartilage samples from our ERp57 KO mice.

In summary, in addition to its role as a chaperone protein in the ER, ERp57 exerts its PDI activity outside of chondrocytes in the ECM to generate functional FN1 fibrils, which are critical for cartilage homeostasis. These results will help to reevaluate the function of PDIs in cartilage tissues and to explore novel targets for skeletal diseases, such as OA or SMD.

### Limitations of the study

We detected ERp57 outside of chondrocytes in the extracellular matrix (ECM) of cartilage, where it binds to FN1-containing structures and contributes to FN1 fibril formation through extracellular PDI activity. A limitation of this study is that it has not yet been fully investigated whether other PDIs can functionally replace ERp57 in this process. To date, only *in vitro* experiments with PDIA1 have been conducted, which showed that C28/I2 ERp57 KO chondrocytes express this PDI. However, PDIA1 is unable to compensate for the loss of ERp57 during FN1 fibril formation. Additional members of the PDI family need to be analyzed in the future. A further limitation of the study concerns the different time periods of the *in vivo* and *in vitro* experiments. The *in vitro* experiments conducted here were short-term analyses lasting up to 7 days. *In vivo*, additional long-term effects may influence FN1 fibril formation and ECM organization that are not present in cell cultures. Furthermore, the ERp57 KO mice used here (ERp57 floxed/floxed, Col2a1 cre) are cartilage-specific ERp57 KO animals. Therefore, secreted ERp57 from neighboring tissues (synovial fibroblasts, meniscus cells, and so forth) may have influenced ECM formation *in vivo*. Finally, we cannot completely rule out that the results of the presented experiments were not influenced at all by the intracellular effects of ERp57. However, we primarily analyzed extracellular ERp57 effects because thiol blockers were used, which cannot easily permeate cell membranes; extracellularly added recombinant ERp57 was not taken up into the cells by endocytosis; and equal amounts of FN1 were present outside the cartilage cells despite ERp57 knockout and ER stress.

## Resource availability

### Lead contact

Requests for further information and resources should be directed to and will be fulfilled by the lead contact, Rita Dreier (dreierr@uni-muenster.de).

### Materials availability

This study did not generate new unique reagents.

### Data and code availability

• All other data reported in this article will be shared with the lead contact upon request.
• This article does not report the original code.
• Any additional information required to reanalyze the data reported in this article is available from the lead contact upon request.

## Acknowledgments

We would like to thank Karin Gäher for excellent technical assistance. The work was supported by the 10.13039/501100001659German Research Foundation (Grants DR455/3-1 and DR455/5-1) to Rita Dreier.

## Author contributions

All authors contributed to the study conception and design. Material preparation, data collection, and analysis were performed by Yvonne Rellmann, Elco Eidhof, Uwe Hansen, Sandra Schulte, and Sina Stücker. The first draft of the article was written by Rita Dreier, and all authors commented on previous versions of the article. All authors have read and approved the final article.

## Declaration of interests

The authors declare no competing interests.

## STAR★Methods

### Key resources table

**Table:** 

REAGENT or RESOURCE	SOURCE	IDENTIFIER
**Antibodies**		

Monoclonal mouse anti-fibronectin	Santa Cruz Biotechnology	Cat#sc-8422; RRID: AB_627598
Polyclonal rabbit anti-fibronectin	Merck	Cat#F3648; RRID: AB_476976
Polyclonal rabbit anti-ERp57	Novus	Cat#NBP1-84796; RRID: AB_11022828
Monoclonal mouse anti-ERp57	Enzo Lifesciences	Cat#ADI-SPA-725; RRID: AB_10618442
Monoclonal mouse anti-Collagen II	Merck	Cat#MAB8887; RRID: AB_2260779
Monoclonal mouse anti-Collagen II	DSHB	Cat#II-II6B3; RRID: AB_528165
Polyclonal rabbit anti-PDI	Santa Cruz Biotechnology	Cat#sc-20132; RRID: AB_653974
Monoclonal rabbit anti-GAPDH (14C10)	Cell Signaling	Cat#2118S; RRID: AB_561053
Donkey anti-rabbit IgG, peroxidase-coupled	Amersham Bioscience	Cat#NA934; RRID: AB_772206
Donkey anti-mouse IgG, peroxidase-coupled	Dianova	Cat#DAB-87152
Goat anti-mouse Alexa Fluor® 488	Thermo Fisher	Cat#A11001; RRID: AB_2534069
Donkey anti-rabbit Alexa Fluor® 568	Thermo Fisher	Cat#A10042; RRID: AB_2534017
Duolink® *In Situ* PLA® Probe Anti-Mouse PLUS	Sigma	Cat#DUO92001; RRID: AB_2810939
Duolink® *In Situ* PLA® Probe Anti-Rabbit MINUS	Sigma	Cat#DUO92005; RRID: AB_2810942

**Chemicals, peptides, and recombinant proteins**		

ERp57-CRISPR/Cas9-knockout plasmid	Santa Cruz Biotechnology	Cat#sc-401497
ERp57-HDR plasmid	Santa Cruz Biotechnology	Cat#sc-401497-HDR
Recombinant human ERp57 protein	Novus	Cat#NBP2-52140
DMEM-High glucose	Merck	Cat#D5796
FCS	PAN Biotech GmbH	Cat#P303031
Sodium pyruvate (Sodium 2-oxopropanoate)	Roth	Cat#8793.1 CAS: 113-24-6
Penicillin/Streptomycin	PAA Laboratories	Cat#P11010
Collagenase B	Roche	Cat#11088831001
Cysteine	Sigma	Cat#C6852 CAS: 7048-04-6
Paraformaldehyde	Serva	Cat# 31628.02 CAS: 30525-89-4
Glutaraldehyde (Pentanedial)	Serva	Cat# 23114.01 CAS:111-30-8
Osmium tetroxide (Tetraoxoosmium)	Roth	Cat#8371.1 CAS: 20816-12-0
Potassium hexacyanoferrate(III)	Sigma	Cat#P8131 CAS: 13746-66-2
Cacodylic acid (Dimethylarsinic acid)	Serva	Cat# 15540.02 CAS:6131-99-3
Propylene oxide ((2R)-2-Methyloxirane, (2S)-2-Methyloxirane)	Merck	Cat# 82320 CAS:75-56-9
Epoxy embedding medium kit	Sigma-Aldrich	Cat#45359
Uranyl acetate (Uranium bis((acetato)-O)dioxo-dihydrate)	Science Services	Cat#E22400 CAS: 6159-44-0
Ethanol	Merck	Cat#107017 CAS: 64-17-5
β-aminopropionitrile fumarate (bis(3-aminopropanenitrile)	Sigma	Cat#A3134 CAS: 2079-89-2
Sodium ascorbate ((5R)-5-[(1S)-1,2-Dihydroxyethyl]-3,4-dihydroxy-5-hydrofuran-2-on)	Sigma	Cat#A4544 CAS: 50-81-7
Monobrom (trimethylammonio) bimanbromid (QBBR)	Merck	Cat#71028 CAS: 71418-45-6
p-Chloromercuriphenylsulphonate (pCMPS)	LGC Standards	Cat#TRC-C367750 CAS: 14110-97-5
Na_2_HPO_4_ (Sodium hydrogen phosphate)	Roth	Cat#27KE.1 CAS: 10028-24-7
MgCl_2_ (Magnesium dichloride)	Merck	Cat#814733 CAS: 7786-30-3
EGTA (3,12-Bis(carboxymethyl)-6,9-dioxa-3,12-diazatetradecane-1,14-dioic acid)	Sigma	Cat#E-4378 CAS: 67-42-5
Nonidet™ P 40, NP-40 (2-[2-(4-Nonylphenoxy)ethoxy]ethanol)	Thermo Scientific	Cat#28324 CAS: 9016-45-9
KCl (Potassium chloride)	Merck	Cat#104936 CAS: 7447-40-7
Acetic acid	Emsure	Cat#100063 CAS: 64-19-7
BSA (Bovine Serum Albumin)	Serva	Cat#11924 CAS: 9048-46-8
Fluoroshield^TM^ + DAPI	Merck	Cat#F6057
Sodium phenylbutyric acid, 4-PBA (Sodium 4-phenylbutanoate)	Santa Cruz Biotechnology	Cat#sc-200652 CAS: 1716-12-7
Thapsigargin ((11S)-7,11-Dihydroxy-12-oxo-6β,12-epoxy-1β,7α,10α-guai-4-ene-2β,3α,8α,10-tetrayl 10-acetate 8-butanoate 3-[(2Z)-2-methylbut-2-enoate] 2-octanoate)	Santa Cruz Biotechnology	Cat# sc-24017 CAS: 67526-95-8
Dithiothreitol, DTT ((2S,3S)-1,4-Bis(sulfanyl)butane-2,3-diol)	Thermo Fisher	Cat# 15397.03 CAS: 3483-12-3
OCT compound	VWR	Cat#361603E
Isopropanol (Propan-2-ol)	Roth	Cat#6752.4 CAS: 67-63-0
Titriplex® III (Disodium;2-[2-[bis(carboxymethyl)amino]ethyl-(carboxylatomethyl)amino]acetate;dihydrate)	Merck	Cat#1.084818 CAS: 6381-92-6
Tris (2-Amino-2-hydroxymethyl-propane-1,3-diol)	MP Biomedicals	Cat#819623 CAS: 77-86-1
Paraffin ROTI®Plast	Roth	Cat#6642.5 CAS: 8002-74-2
ROTI®Histol ((2Z,6E)-2,6-dimethyl-10-methylidenedodeca-2,6,11-trienal)	Roth	Cat#6640.4 CAS: 8028-48-6
Protease XXIV	Sigma	Cat#P8038 CAS: 9014-01-1
Hyaluronidase	Sigma	Cat#H3506 CAS: 37326-33-3
Chondroitinase ABC	Sigma	Cat#C3667 CAS: 9024-13-9
HCl (Hydrochloric acid)	Merck	Cat#1.00314.2500 CAS: 7647-01-0
Sodium acetate	Merck	Cat#1.06268.1000 CAS: 127-09-3
Tween-20 (2-(2-[3,4-bis(2-Hydroxyethoxy)oxolan-2-yl]-2-(2-Hydroxyethoxy)ethoxy)ethyl-Dodecanoat)	Sigma	Cat#P1379 CAS: 9005-64-5
Milk powder	Millipore	Cat#70166
Clarity Western ECL Substrate	BioRad	Cat#1705061
SuperSignal™ West Femto Maximum Sensitivity Substrate	Thermo Scientific	Cat# 34094

**Critical commercial assays**		

Duolink® *In Situ* Proximity Ligation Detection Reagent	Sigma	Cat#DUO92008

**Experimental models: Cell lines**		

C28/I2 WT cells	Goldring et al.^63^	RRID: CVCL_0187
C28/I2 ERp57 KO cells	Rellmann et al.^38^	N/A

**Experimental models: Organisms/strains**		

ERp57*fl/fl* mice	Garbi et al.^64^	N/A
Col2a1-cre mice	Sakai et al.^65^	N/A
ERp57fl/fl-Col2a1-cre	Linz et al.^34^	N/A

**Software and algorithms**		

ZEN 3.6 blue edition	Carl Zeiss AG	ZEISS ZEN Mikroskopie-Software
GraphPad Prism (Version 6)	GraphPad Software Inc.	Prism - GraphPad
ImageJ 1.54f	Schneider et al.^66^	http://imagej.org

**Other**		

Ultramicrotome Leica Ultracut EM UC7	Leica	RRID: SCR_016694
Philips EM-410	Philips	N/A
Microtome Epredia HM 355S	Epredia	RRID:SCR_026146
Cryostat Leica CM1950	Leica	RRID:SCR_018061
Zeiss Laser scanning confocal microscope LSM 900	Carl Zeiss AG	RRID: SCR_022263
Removable 8-well chamber slides	Ibidi GmbH	Cat#80841
Blotting transfer system BioRad Trans-Blot Turbo	BioRad Laboratories	RRID:SCR_023156
Chemiluminescent imager Fusion-SL	Peqlab	N/A

### Experimental model and study participant details

#### Cartilage-specific ERp57 knockout mice (ERp57 KO)

ERp57 KO mice were generated by breeding of *PDIA3*^fl/fl^ C57BL6 mice with *Col2a1*-CreC57BL6 mice as described.^34^ To gain WT (*PDIA3*^fl/fl^) and ERp57 KO (*PDIA3*^fl/fl^-*Col2a1*-cre) littermates, homozygous *PDIA3* floxed *Col2a1*-cre-positive mice were mated with homozygous *PDIA3* floxed *Col2a1*-cre-negative mice. In compliance with the German federal law for animal protection under the control of the North Rhine-Westphalia State Agency for Nature, Environment and Consumer Protection (LANUV, NRW, AZ 81-02.05.50.22.015) the animals were kept under pathogen-free conditions and supplied with food and water *ad libitum* in a 12-hour light/dark cycle. For transmission electron microscopy analysis, 4 WT and 4 ERp57 KO mice (2 males and 2 females each) were sacrificed at the age of 18 weeks. To analyze the ECM of primary chondrocytes of WT and ERp57 KO animals, knee and hip cartilage was dissected from 4 WT and 4 ERp57 KO newborn mice (2 males and 2 females each). For all experiments with mice or with chondrocytes isolated of murine articular cartilage, equal numbers of male and female mice were used to exclude any influence of sex on the experimental results. It has previously been observed that estradiol inhibits ER stress-induced apoptosis in chondrocytes and contributes to reduced osteoarthritic cartilage degeneration in female mice.^55^ Details on experiments with animal tissue or cells are listed in the ARRIVE checklist.

#### C28/I2 WT cells and ERp57 knockout C28/I2 cells

C28/I2 WT chondrocytes and CRISPR/Cas9-generated ERp57 knockout C28/I2 cells (ERp57 KO)^38^ were cultured at 37°C, 5% CO_2_, and 100% humidity in DMEM (D579, Merck, Darmstadt, Germany) supplemented with 10% FCS, 1% sodium pyruvate, 100 units/ml penicillin and 100 μg/ml streptomycin. The immortalized chondrocyte cell line C28/I2 originated from cells isolated from rib cartilage of a 15-year-old female.^63^ It was not specifically authenticated for this study but was tested for mycoplasma contamination using PCR analysis. To verify the chondrocyte phenotype regular controls for proteoglycan staining (Alcian blue) and protein expression of Col II, Col I, Col VI, Col X were performed.

### Method details

#### Transmission electron microscopic analysis (TEM)

WT and ERp57 KO mice (n = 4 for each genotype) were sacrificed at the age of 18 weeks. Mice of this age were used, as they are fully grown, but do not yet show any arthritic changes in the cartilage.^34^^,^^35^ Knee joint cartilage was dissected and fixed overnight in 2% (v/v) paraformaldehyde (PFA) (Serva, Heidelberg, Germany) and 2.5% (v/v) glutaraldehyde (Serva, Heidelberg, Germany) in 100 mM phosphate buffer (PBS), pH 7.4 at 4°C for morphological analysis. Tissue samples were postfixed in 0.5% (v/v) osmium tetroxide (Roth, Karlsruhe, Germany) and 1% (w/v) potassium hexacyanoferrate (III) (Sigma-Aldrich, St. Louis, MO, USA) in 0.1 M cacodylate buffer (Serva, Heidelberg, Germany) for 2 h at 4°C, followed by washing with distilled water. After dehydration in an ascending ethanol series from 30 to 100%, specimens were incubated two times in propylene oxide (Merck, Darmstadt, Germany) each for 15 min and embedded in Epon (Sigma-Aldrich, St. Louis, MO, USA) using flat embedding molds. Ultrathin sections were cut with an ultramicrotome (Leica Ultracut EM UC7), collected on copper grids, and negatively stained with 2% uranyl acetate (Science Services, Munich, Germany) for 15 min.

To analyze the ECM of primary chondrocytes of WT and ERp57 KO animals, knee and hip cartilage was carefully dissected from newborn mice (4 animals per genotype) and incubated overnight in DMEM supplemented with 1 mg/ml Collagenase B (Roche, Mannheim, Germany) and 1 mM cysteine. 400,000 cells were pipetted in a 20-μl droplet in DMEM into the well of a 24-well plate and kept for 3 hours at 37°C, 5% CO_2_ and 100% humidity in an incubator to form micromass cultures.^67^ For these experiments, newborn mice were used to obtain sufficient cells for the appropriate number of micromass cultures. The cells were then cultured for 7 days in 1 ml DMEM containing 60 mg/ml β-aminopropionitrile fumarate, 25 mg/ml sodium ascorbate, 1 mM cysteine, 1 mM pyruvate (ABCP), at 37°C, 5% CO_2_ and 100% humidity. To analyze the ECM by TEM, the micromasses (≥10 micromasses per genotype) were fixed overnight with 2% (v/v) PFA and 2.5% (v/v) glutaraldehyde in 100 mM cacodylate buffer (pH 7.4) at 4°C and washed in PBS. Postfixation, dehydration, embedding and cutting was performed as mentioned above. The sections were negatively stained with 2% uranyl acetate (Science Services, Munich, Germany) for 10 min. To exclude different cell numbers of WT and KO cells in the analyzed micromasses after the culture period of 7 days, cells were counted in images of all analyzed WT and ERp57 KO samples. For both types of specimens, a Phillips EM-410 electron microscope was used to take electron micrographs at 60 kV using imaging plates (Ditabis, Pforzheim, Germany). To quantify the density of the fibrillar network, several randomly selected fields on EM images of WT and ERp57 KO chondrocyte micromass cultures were analyzed with ImageJ 1.54f (http://imageJ.org/).^66^

#### Immunofluorescence analysis of C28/I2 cells

20,000 C28/I2 WT and ERp57 KO cells were seeded per well in DMEM supplemented with 10% FCS, 1% sodium pyruvate, 100 units/ml penicillin and 100 μg streptomycin + ABCP on 8-well IBIDI slides with a removable chamber (80841, Ibidi GmbH, Gräfelfing, Germany). The cells were cultured for 72 hours at 37°C, 5% CO_2_ and 100% humidity. To analyze extracellular disulfide bridge formation or the role of extracellular ERp57, cultures were supplemented during the entire culture period with 60 and 300 μM Monobrom (trimethylammonio) bimanbromid (QBBR) (Merck, Darmstadt, Germany), 1.5 and 15 μM p-Cholromercuriphenylsulphonate (pCMPS) (LGC Standards, Wesel, Germany) or 0.1 μM active recombinant human ERp57 protein (NBP2-52140, Novus, Centennial, CO, USA), respectively. To assess cellular uptake of ERp57, ERp57 KO cells were cultured in conditioned media from ERp57 WT cells or treated with 0.1 μM recombinant human ERp57 protein (NBP2-52140, Novus, Centennial, CO, USA). ER stress was induced by adding 1mM thapsigargin (Tg) (Santa Cruz, sc-24017A) to the culture medium. To reduce ER stress, cells were treated with 1mM 4-PBA (Santa Cruz, sc-200652). Some samples were decellularized to analyze the ECM without interfering fluorescent signals from the cells. The cells were washed once with PBS, two times with 100 nM Na_2_HPO_4_ (pH 9.6) + 2 mM MgCl_2_ + 2 mM EGTA and then incubated for 15 min at 37°C in lysis buffer containing 8 mM Na_2_HPO_4_ (pH 9.6) + 1 % NP-40. A second incubation with lysis buffer for additional 40-60 min at 37°C was performed before washing with 300 mM KCl + 10 mM Na_2_HPO_4_ (pH 7.5) (2 times) and double distilled H_2_O (4 times).

Before immunofluorescence staining, the cells and/or the matrix were fixed with 1 % para-formaldehyde in PBS at pH 7.4 (PFA/PBS) for 10 min at room temperature (RT) and washed twice for 5 min with PBS. Samples were incubated in ethanol:acetic acid (2:1) for 5 min at -20°C as additional fixation and/or permeabilization step and washed with PBS (3 times, 5 min). Unspecific protein binding was blocked for 1 hour with 5 % BSA in PBS at RT, and the samples were subsequently incubated overnight at 4°C with the primary antibody diluted in 2 % BSA/PBS. To analyze FN1 in the newly generated ECM, antibodies against fibronectin (sc-8422, monoclonal, from mouse, Santa Cruz, Dallas, USA, 1:50 or F3648, polyclonal, from rabbit, Merck Darmstadt, Germany, 1:200) were used. To analyze PDI, extracellular ERp57 or Col II-containing fibrils, a rabbit polyclonal PDI antibody (sc-20132, Santa Cruz Biotechnology, USA, 1:50), a rabbit polyclonal ERp57 antibody (NBP1-84796, Novus, Centennial, USA,1:200) or monoclonal mouse Collagen II antibodies (MAB8887, Merck Darmstadt, Germany, 1:200, or DSHB II6B3, 1:200) were applied. After overnight incubation with the primary antibodies, the samples were rinsed with PBS (3 times for 5 min) and then incubated with Alexa Fluor® 488-labelled goat anti-mouse secondary antibody (Invitrogen Thermo Fisher, Waltham, USA, A11001,1:500) or Alexa Fluor® 568-labelled donkey anti-rabbit secondary antibody (Thermo Fisher, Waltham, USA Invitrogen, A10042, 1:500) in 2 % BSA/PBS for 1 hour at RT in the dark. After three washing steps with PBS for 5 min, the silicone chambers of the IBIDI slides were removed and the slides were mounted with Fluoroshield^TM^ + DAPI (F6057, Merck KGaA, Darmstadt, Germany). Images were taken using a Zeiss LSM900 inverted confocal microscope (CLSM, Carl Zeiss AG, Oberkochen, Germany) equipped with Zen version 3.6 blue edition.

#### *In situ* proximity ligation assay

20,000 C28/I2 WT and ERp57 KO cells were seeded per well in DMEM supplemented with 10 % FCS, 1 % sodium pyruvate, 100 units/ml penicillin and 100 μg streptomycin + ABCP on 8-well IBIDI slides with a removable chamber (80841, Ibidi GmbH, Gräfelfing, Germany). The cells were cultured for 72 hours at 37°C, 5 % CO_2_ and 100 % humidity. Some cultures were subsequently incubated with 10 mM DTT (Thermo Fisher Scientific, Darmstadt, Germany) for 15 min at 37°C. All samples were then decellularized, fixed, blocked and incubated overnight with primary antibodies against fibronectin (sc-8422, monoclonal, from mouse, 1:50 Santa Cruz, Dallas, USA) or Col II (MAB8887, Merck Darmstadt, Germany, 1:200) and ERp57 (Novus, Centennial, USA, NBP1-84796, 1:200). Subsequently, the specimens were subjected to Duolink® *in situ* fluorescence detection according to the manufacturer's instructions. Briefly, slides were incubated with secondary antibodies conjugated to oligonucleotides (DUO92001 and DUO 92005, Merck KGaA, Darmstadt, Deutschland). Circularization and ligation of the oligonucleotides was followed by an amplification step with fluorescent oligonucleotides. Negative controls included WT cells expressing both target proteins with either anti-FN (FN Ctrl), anti-ERp57 (ERp57 Ctrl) or both primary antibodies omitted (negative control, NC). ERp57 KO cells, expressing only Col II but no ERp57 served as an additional control. Slides were mounted with Fluoroshield^TM^ + DAPI mounting medium and evaluated with an LSM 900 confocal microscope (CLSM, Carl Zeiss AG, Oberkochen, Germany).

#### Immunoblot analysis

Micromasses of primary chondrocytes were generated as described above^67^ and cultured for 72 h in DMEM + 1 % sodium pyruvate, 100 units/ml penicillin and 100 μg streptomycin + ABCP containing 10 % FCS and then for 48 h under similar, but serum-free conditions at 37°C, 5 % CO_2_ and 100 % humidity. Monolayer cultures of C28/I2 cells were performed as described above (see immunofluorescence analysis of C28/I2 cells). Micromasses and cell monolayers were lysed and boiled in SDS sample buffer (1 mM EDTA, 1 % SDS, 10 % glycerol, 0.01 % bromophenol blue, 10mM Tris/HCl, pH 8.0). TCA-precipitated media samples and lysed cell samples were run on a 4.5-15 % SDS-PAGE gel under reducing or non-reducing conditions. Proteins were blotted on a nitrocellulose membrane using a western blotting transfer system (BioRad Transblot Turbo). Membranes were blocked in blocking buffer (5 % skim milk and 1 % BSA in tris-buffered saline with 0.1 % Tween 20 (TBS-T)) and incubated with a monoclonal mouse anti-fibronectin (sc-8422, Santa Cruz, 1:1000), anti-ERp57 (ADI-SPA-725, Enzo Lifescience, 1:1000) or rabbit anti-GAPDH (14C10, Cell Signaling, 1:1000) antibody overnight at 4°C. After washing in TBS-T, membranes were incubated with peroxidase-coupled donkey anti-mouse (DAB-87152, Dianova 1:1000) or anti-rabbit (NA934, Amersham Bioscience, 1:10000) secondary antibodies for 1 hour at room temperature. Signals were visualized with an enhanced chemiluminescent substrate (Clarity Western ECL Substrate, BioRad or SuperSignal™ West Femto Maximum Sensitivity Substrate, Thermo Scientific) and detected by chemiluminescence imaging (Peqlab Fusion-SL 3500.WL).

#### Histological staining of knee joints

WT and ERp57 KO mice were sacrificed at the age of 14 weeks and knee joints were dissected for paraffin or cryo embedding. Knee joints were embedded in OCT compound mounting medium (361603E, VWR, Darmstadt, Germany), frozen in liquid nitrogen and stored at - 80°C. Sections of 4.5 μm thickness were cut on a cryostat (CM1950, Leica, Wetzlar, Germany). For paraffin embedding, knees were fixed in 4 % PFA overnight and washed in distilled water. Knee joints were decalcified in 20 % (w/v) ethylene diamine tetraacetic acid (EDTA) (Titriplex® III, Merck, Darmstadt, Germany) with 66 g/L Tris for 2 weeks with the solution being changed every 2 days. Decalcified joints were dehydrated in a series of ethanol and isopropanol and embedded in paraffin (ROTI®Plast 6642.5, Roth, Karlsruhe, Germany). Paraffin embedded knee joints were cut into sections of 4.5 μm thickness on a microtome (HM 355S, Epredia, Dreieich, Germany). For collagen II staining, paraffin sections were deparaffinized. Antigen retrieval was performed by incubation at 37°C with protease XXIV (0.05 % in PBS, pH7.4) for 10 min followed by chondroitinase ABC (0.2 U/ml) in 50 mM Tris, 60 mM sodium acetate and 0.01 % (w/v) BSA for 90 min, and 1 % (w/v) pepsin in 0.01 M HCl for 10 min. For fibronectin staining, cryo-sections were thawed, rinsed in PBS and fixed in 1 % PFA. Fixed sections were treated with protease XXIV (0.05 % in PBS, pH 7.4) for 10 min and hyaluronidase (0.1 % in sodium acetate, pH 6.0) for 90 min at 37°C. Unspecific protein binding was blocked for 1 hour with 5 % BSA in PBS at RT. Sections were stained using rabbit polyclonal fibronectin antibody (F3648, Merck, Darmstadt, Germany 1:100) or monoclonal mouse Collagen II antibody (MAB8887, Merck, Darmstadt, Germany, 1:500) and the respective secondary antibodies as described above. Slides were mounted with Fluoroshield^TM^ + DAPI (F6057, Merck KGaA, Darmstadt, Germany) and evaluated with an LSM 900 confocal microscope (CLSM, Carl Zeiss AG, Oberkochen, Germany).

### Quantification and statistical analysis

All images were analyzed with ImageJ 1.54f (http://imageJ.org).^66^ The density of the fibrillar network was quantified on several randomly selected fields on EM images of WT and ERp57 cartilage or micromass cultures. Mean staining intensities of FN1 and Col II networks and PLA signals were measured on immunofluorescent images. The intensity of chemiluminescence signals on immunoblots was densitometrically evaluated. For the statistical analysis, data are presented as means ± SD. Parametric (Student t-test) tests, ordinary one-way ANOVA or two-way ANOVA (analysis of variants), both with Tukey’s post-hoc test, were performed using GraphPad Prism, V.6.0h (GraphPad Software Inc., San Diego, USA), with p < 0.05 determining the primary level of significance. The sample size and the method of data presentation and statistical analysis for each graph is reported in the respective figure legend.

## Supplemental information

Document S1. Figures S1–S13 and Table S1

Data S1. Arrive checklist
