# Supplementary material for: Extracellular ERp57 promotes fibronectin fibril formation during matrix assembly of articular cartilage
Source: iScience. 2025 Nov 13;28(12):114046. doi: 10.1016/j.isci.2025.114046 (PMC12702194; doi:10.1016/j.isci.2025.114046)
Supplement: Document S1. Figures S1–S13 and Table S1 [file mmc1.pdf]

iScience, Volume *28*

Supplemental information

Extracellular ERp57 promotes fibronectin fibril formation during matrix assembly of articular cartilage

Yvonne Rellmann, Elco Eidhof, Uwe Hansen, Sandra Schulte, Sina Stücker, Thomas Pap, and Rita Dreier

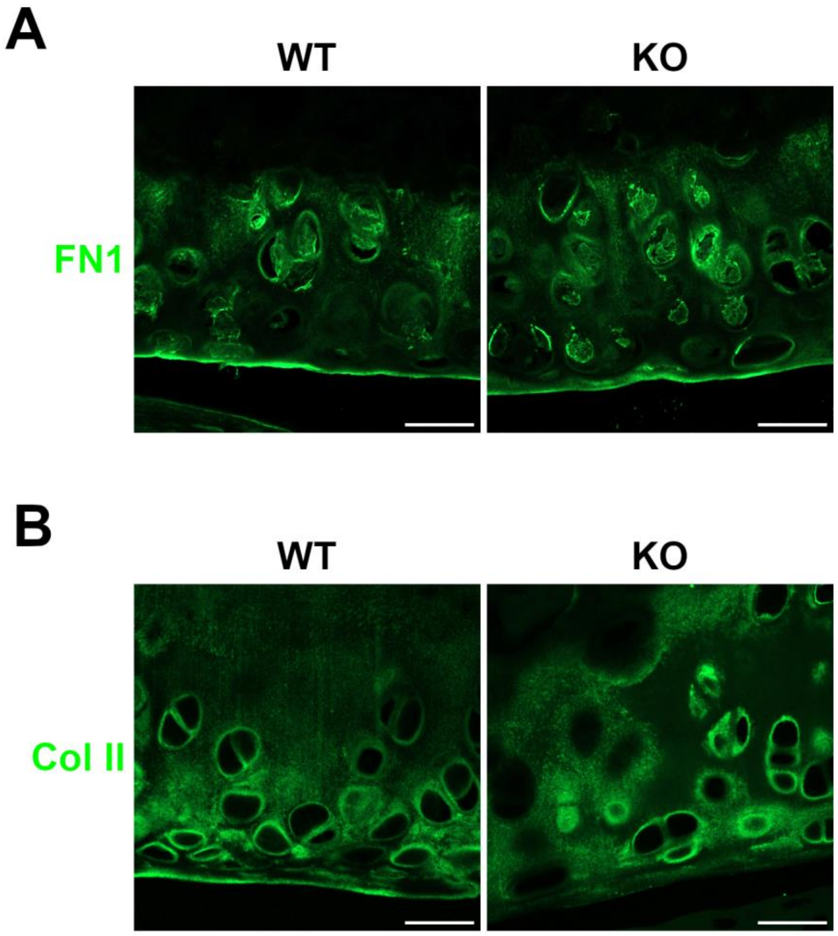

Fig. S1: FN1 and Col II immunofluorescence staining of WT and ERp57 KO articular knee cartilage. FN1 was analyzed on frozen tissue sections (A) and Col II staining was performed on paraffin sections (B) of 14-week-old mice. Both genotypes showed similar amounts of both ECM proteins with a comparable distribution in the tissues. However, the resolution of this method is insufficient to depict the structural differences between WT and KO tissue evident in TEM images, and to distinguish between soluble FN1 and short, thin FN1 fibrils found in KO tissues and large, thick fibrillar structures generated by ERp57 activity in WT cartilage. Furthermore, the used mouse model is cartilage-specific (ERp57 floxed-Col2a1 cre) and thus other cells in the joint, such as synovial fibroblasts or myocytes, could produce and secrete active ERp57. Scale bar = 20 µm.

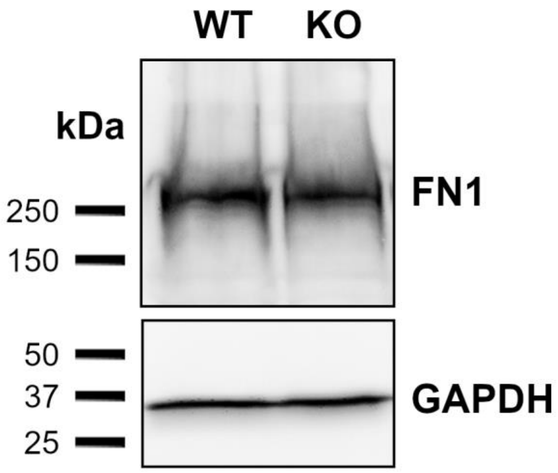

Fig. S2: Immunoblot of lysates of micromass cultures generated from isolated primary chondrocytes of newborn WT and ERp57 KO mice. Fibronectin (FN1) was detected in equal amounts in WT and ERp57 KO samples. The corresponding signals of glycerinaldehyde-3-phosphate dehydrogenase (GAPDH) were shown as internal control.

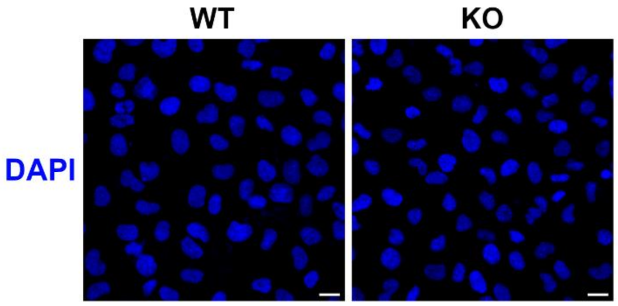

Fig. S3: DAPI staining of fixed C28/I2 wildtype (WT) and C28/I2 ERp57 knockout (KO) chondrocytes. The staining confirmed an equally high vitality and comparable proliferation of cells of both genotypes in cell culture experiments. Scale bar = 20 µm.

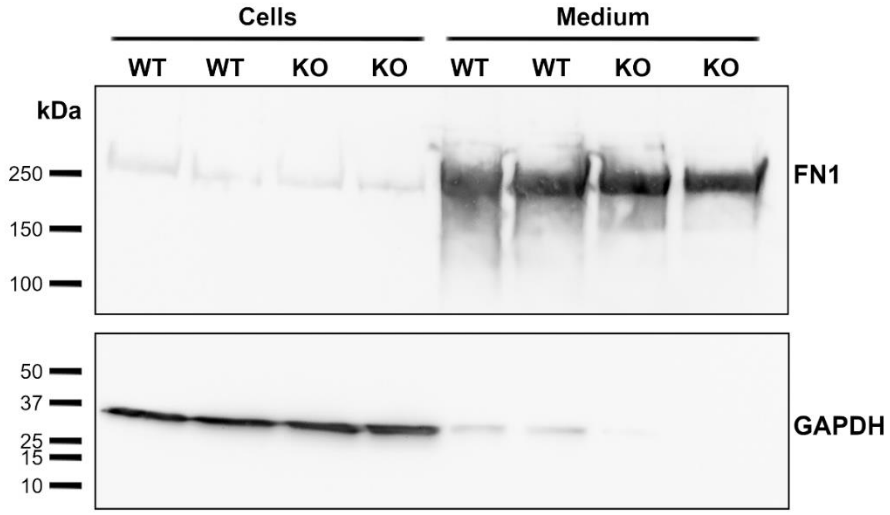

Fig. S4: Immunoblot of cell lysates and culture media of C28/I2 wildtype (WT) and C28/I2 ERp57 knockout (KO) cells. Fibronectin (FN1) was secreted in comparable amounts into the medium of WT and KO cells. The corresponding signals of glycerinaldehyde-3-phosphat-dehydrogenase (GAPDH) are shown as internal control.

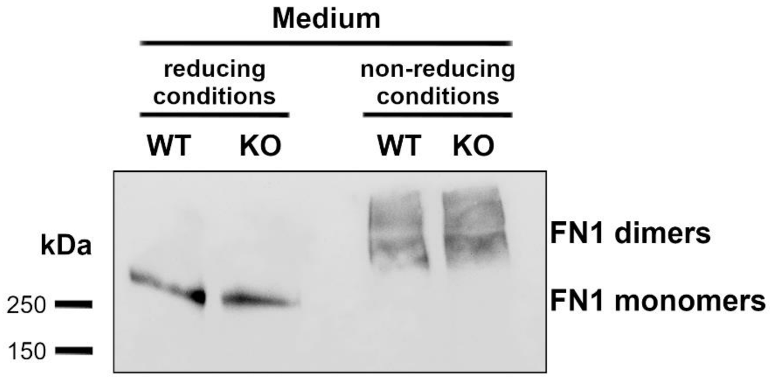

Fig. S5: Immunoblot of FN1 in the culture medium of C28/I2 WT and C28/I2 ERp57 knockout (KO) cells under reducing (+ ß-mercaptoethanol) and non-reducing (- ß-mercaptoethanol) conditions. Under reducing conditions, FN1 was detectable in its monomeric form in equal amounts in WT and KO samples. Under non-reducing conditions, dimeric FN1 was observed in comparable amounts in both genotypes. Thus, the loss of ERp57 in the KO samples did not affect the levels of monomeric or dimeric FN1.

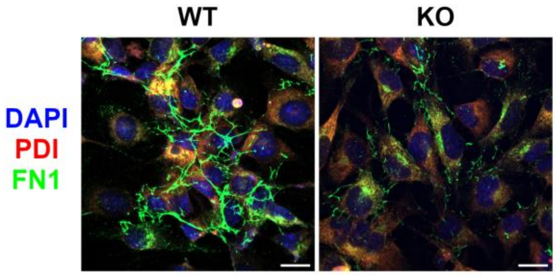

Fig. S6: PDI and FN1 immunofluorescence staining of C28/I2 WT and C28/I2 ERp57 KO chondrocytes. The nuclei are stained with DAPI (blue). Both genotypes show similar amounts of PDI (red staining), but reduced FN1 fibrillogenesis (green staining) in C28/I2 ERp57 KO chondrocytes compared to WT cells. Therefore, PDI is likely not involved in FN1 fibrillogenesis and cannot compensate for the loss of ERp57 in KO cells. Scale bar = 20 µm.

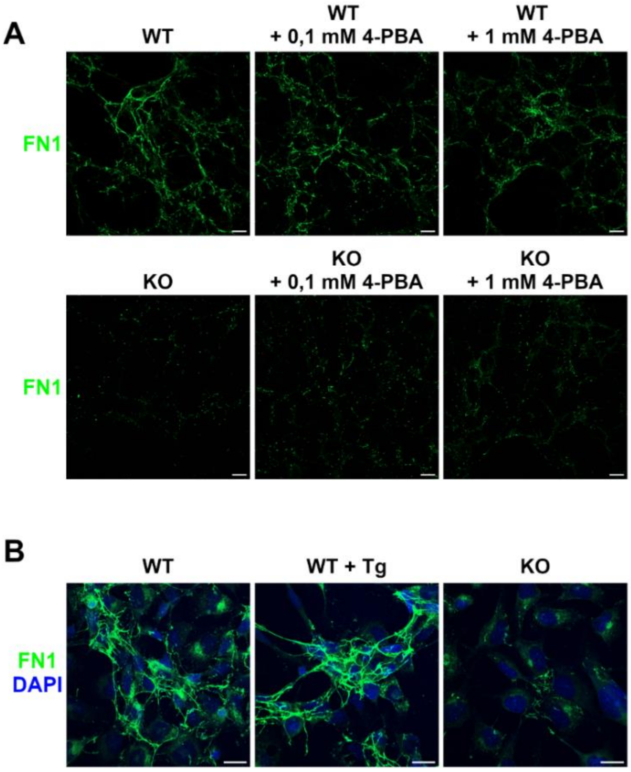

Fig. S7: Immunofluorescence-analysis of FN1 fibrils produced by C28/I2 WT and C28/I2 ERp57 knockout (KO) cells after culture in presence of the chemical chaperone 4-PBA and the ER stress inductor Thapsigargin. 0.1 mM or 1 mM 4-PBA had no effect on the formation of FN1 fibrils in cultures of WT or KO cells, in which FN1 fibrils occur only in reduced amounts (A). WT cells treated with Thapsigargin showed no difference in FN1 fibril formation compared to untreated cells (B). Accordingly, FN1 fibril formation is neither affected by the chemical chaperone, nor the ER stress inductor, which supports the assumption, that the reduction of FN1 fibrils in KO chondrocytes is not based on increased ER stress due to the loss of intracellular ERp57. Nuclei were stained with DAPI. Scale bar = 20 µm.

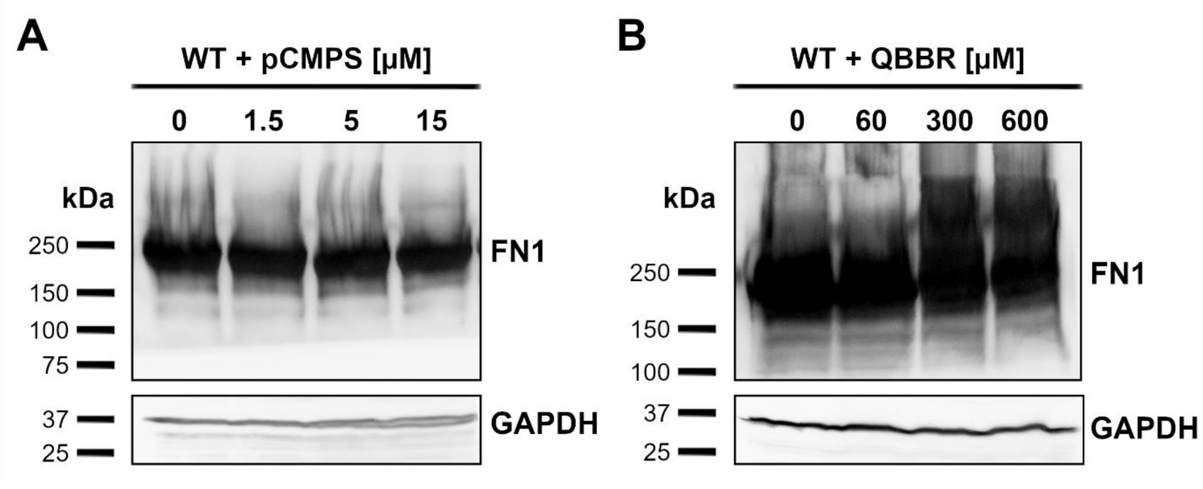

Fig. S8: Immunoblot of culture media of C28/I2 wildtype (WT) cells after culture in presence of thiol blocking agents. During the culture period of 72 h fibronectin (FN1) is secreted in comparable amounts into the medium of WT cells, regardless of the added thiol blocking agents p-Chloromercuriphenylsulfonate (pCMPS) (A) and Monobromo (trimethylammonio) bimanbromide (QBBR) (B). The signals of glycerinaldehyde-3-phosphat-dehydrogenase (GAPDH) from the corresponding cell lysates are shown as internal controls.

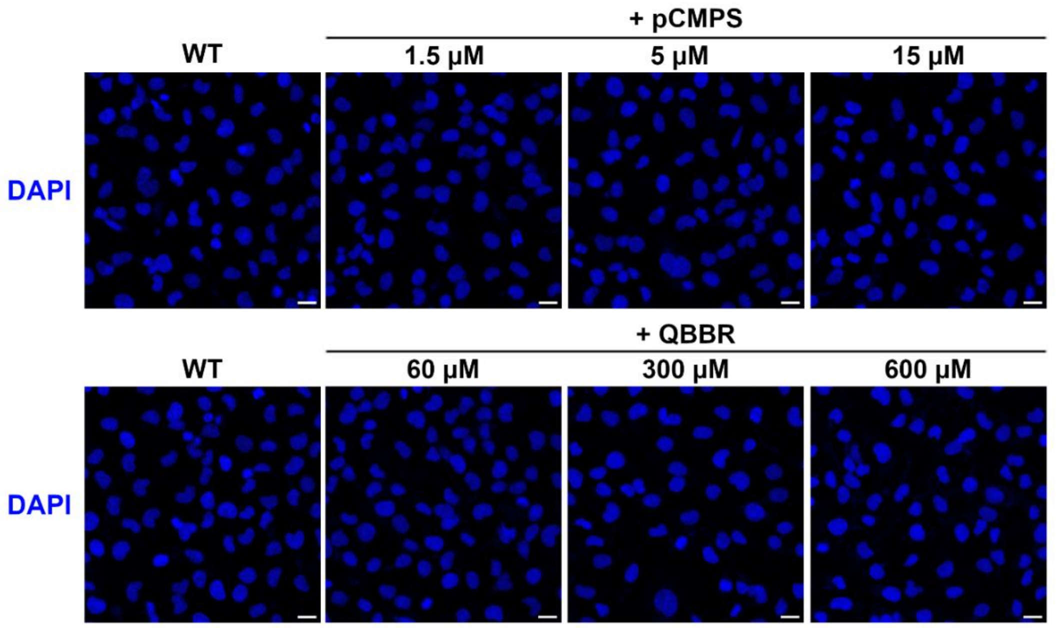

Fig. S9: DAPI staining of C28/I2 WT chondrocytes after culture in presence of thiol blocking agents. The staining confirms equally high vitality and comparable proliferation of the cells after cultivation in presence of different concentrations of p-Chloromercuriphenylsulfonate (pCMPS) and Monobromo (trimethylammonio) bimanbromide (QBBR). Scale bar = 20 µm.

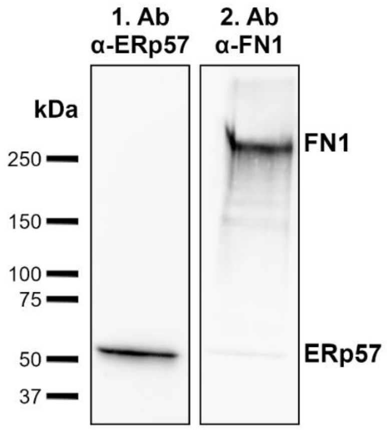

Fig. S10: Immunoblot of a C28/I2 WT cell lysate to determine the specificity of the ERp57 antibody (Ab). The blot was first incubated with an α-ERp57 antibody, the corresponding secondary antibody and the signal was detected (left lane). The right lane shows the result of the subsequent incubation and detection with an α-FN1 antibody and its corresponding secondary antibody on the same blot. In addition to the strong FN1 signal, the signal of the previously detected ERp57 protein is still slightly visible. Together, this result shows that the α-ERp57 antibody is specific for ERp57 and shows no cross-reactivity for FN1.

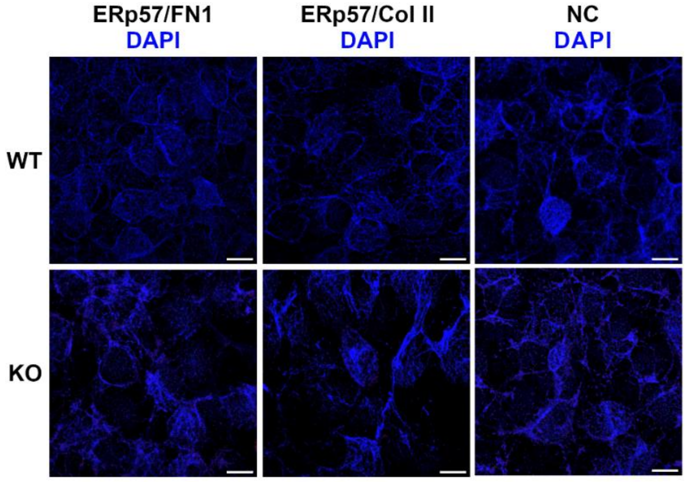

Fig. S11: DAPI staining of decellularized C28/I2 WT and C28/I2 ERp57 knockout (KO) samples. The staining confirms equal amounts of matrix used for proximity ligation assay (PLA) experiments. NC = Negative control. Scale bar = 20 µm.

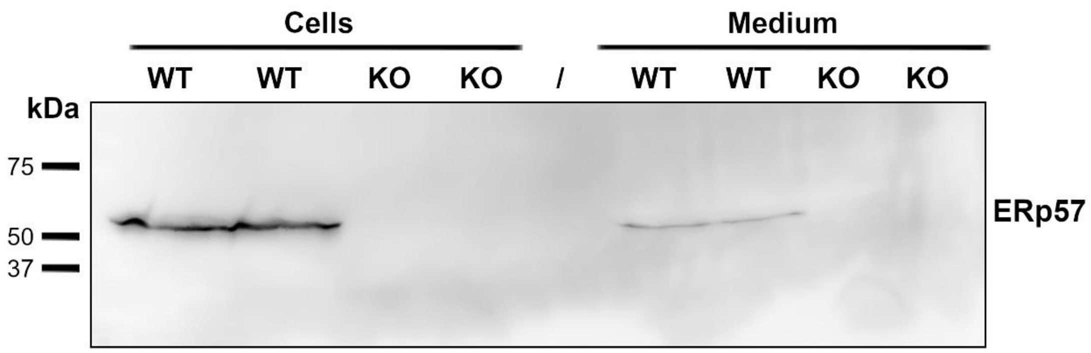

Fig. S12: Immunoblot of C28/I2 WT and C28/I2 ERp57 KO chondrocytes samples to detect the relative amount of ERp57 in cells and cell culture media. The signal intensities of the cell lysates and culture media were calculated using ImageJ. The sum of both intensities was set to 100 %. Approximately 9 % of the total amount of the ERp57 protein is secreted into the medium. As expected, the KO cells show no ERp57 signal.

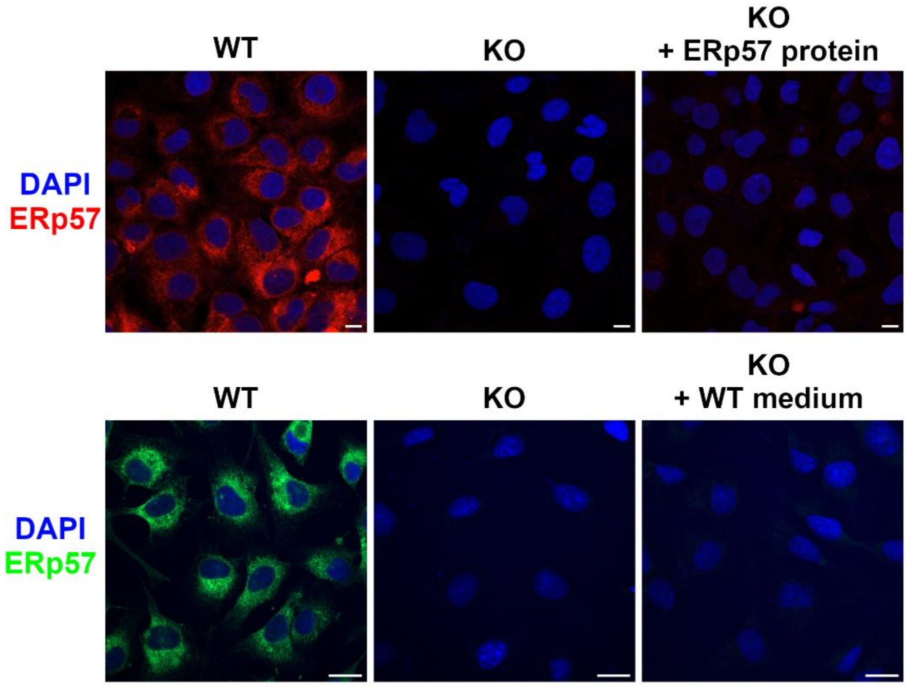

Fig. S13: ERp57 staining of C28/I2 WT and C28/I2 ERp57 KO chondrocytes to analyze possible ERp57 uptake. ERp57 can be detected in WT cells, especially near the nuclei (stained with DAPI) in the endoplasmic reticulum, while the KO cells show no ERp57 signal, as expected. Even after cultivation of KO cells with 0.1 µM ERp57 protein or transfer of conditioned medium from WT to KO cells, no ERp57 signal was evident in the cells. Thus, no endocytosis of ERp57 into cells was detected. Scale bar = 20 µm.

Supplementary table 1: Study design and samples

Readout	Method	Figure	Groups	Sample Size	Comments
In vivo analysis of articular cartilage in WT and ERp57 KO mice	Transmission electron microscopy (TEM)	1	18-week-old WT and ERp57KO mice	4 WT and 4 ERp57 KO mice	8 pictures per genotype
In vitro analysis of fibril formation of WT and ERp57 KO chondrocytes	Transmission electron microscopy (TEM)	2	Chondrocytes isolated from newborn WT and ERp57cKO mice	4 WT and 4 ERp57 cKO mice	>10 micromasses per genotype
